# Adjuvant imatinib for patients with high-risk gastrointestinal stromal tumors: a retrospective cohort study

**DOI:** 10.1038/s41598-017-17266-5

**Published:** 2017-12-04

**Authors:** Rui Zhao, Yong Wang, Yuqian Huang, Yaping Cui, Lin Xia, Yi Chen, Wen Zhuang, Yong Zhou, Xiaoting Wu

**Affiliations:** Department of Gastrointestinal Surgery, West China Hospital, Sichuan University, 37 Guo Xue Rd, Chengdu, 610041 Sichuan Province China

## Abstract

The duration of adjuvant imatinib for high-risk patients with gastrointestinal stromal tumors (GISTs) is still controversial. Therefore, we retrospectively analyzed the data of high-risk patients with GISTs to investigate the appropriate duration. All 185 patients were divided into 4 groups: <1 year (Group A), 1–2 years (Group B), 2–3 years (Group C) and >3 years (Group D). The mean recurrence-free survival (RFS) in Groups A, B, and C were 44.3, 62.1, and 86.8 months, respectively (*P* < 0.001); the mean overall survival (OS) in Groups A, B and C was 75.2, 88.1, and 94.7 months, respectively (*P* = 0.009). The 5-year RFS in Groups A, B, C, and D was 15%, 26%, 83%, and 100%, respectively (*P* < 0.001); and the 5-year OS was 64%, 88%, 88%, and 100%, respectively (*P* < 0.001). The greatest impact on unfavorable outcomes was the tumor mitotic rate (HR, 2.01, 95% CI, 1.38–2.94; *P* < 0.001). Duration of adjuvant imatinib was the only favorable factor (HR, −0.95, 95% CI, 0.93–0.97; *P* < 0.001). For high-risk patients with high tumor size or mitotic rate, or non-gastric GISTs, we recommend that more than 3 years of adjuvant imatinib is feasible.

## Introduction

Gastrointestinal stromal tumors (GISTs) are the most common gastrointestinal soft tissue malignancies^1^. In the past, the outcomes of patients suffering from GISTs were unfavorable because of the lack of response to other interventions except surgery, such as radiotherapy and chemotherapy. Complete surgical resection was the standard treatment for localized, primary GISTs, but approximately 40–50% have disease recurrence^2^. The study by Hirota and colleagues was the first to find that the activating mutations in *KIT* resulted in GISTs, significantly altering the biological understanding and management of this disease^3^. Now, we know that approximately 85% of GISTs contain an activating mutation in the *KIT* proto-oncogene, whereas 5–10% can have a mutation in the gene encoding platelet-derived growth factor receptor α (*PDGFRα*)^4–8^. Imatinib can inhibit activation of *KIT* and *PDGFRα* to treat GISTs. In 2000, imatinib was first used to treat metastatic GISTs effectively and has since been confirmed by successive research^9–13^. Imatinib has significantly changed the therapy of GISTs, and it is recommended as the standard first-line agent in the treatment of GISTs^14,15^. Adjuvant imatinib is the key treatment for postoperative high-risk patients with GISTs to decrease relapse^16^. Three large, randomized phase III trials have clearly demonstrated the benefit of adjuvant imatinib in high-risk patients with GISTs^14,17,18^. Consensus has been reached that adjuvant imatinib is necessary for high-risk patients with GISTs after surgery. However, there are still a number of controversies in the management of GISTs^19,20^. Some guidelines recommend that^3^ years of adjuvant imatinib for high-risk patients is effective to decrease recurrence^21–24^. The primary evidence is based upon a randomized trial (SSGXVIII/AIO) that showed that postoperative imatinib administered for 36 months can improve recurrence-free survival (RFS) and overall survival (OS) compared to 12 months for high-risk patients with GISTs^18^. However, before the randomized trial (SSGXVIII/AIO), two randomized trials recommended that adjuvant imatinib for 1 year could be effective to decrease recurrence (Z9001 ACOSOG) and that adjuvant imatinib for 2 years could prolong RFS for 1 year^14,25,26^. Based on three large, randomized trials, the longer duration of adjuvant imatinib was better. Is a 3-year duration of adjuvant imatinib sufficient for high-risk patients with GISTs? The relevant research is sparse, and to date, little prospective data available on the problem have been published. Therefore, our study was conducted to retrospectively analyze the clinical data of high-risk patients with GISTs after complete resection to investigate the appropriate duration of adjuvant imatinib.

## Results

### Characteristics

The 185 patients included 108 males and 77 females, and the mean age was 54.9 ± 12.0 years. The most frequent location of the primary tumor was the stomach (n = 124, 67.0%), followed by the small intestine (n = 49, 26.5%) and colorectal tract (n = 12, 6.5%). The baseline characteristics of the patients are shown in Table 1. We found that risk factors (tumor size, tumor site, tumor mitotic and GI bleeding) in the four groups had insignificant differences (Table 1). Among the 11 patients who did not receive imatinib for 12 months, 5 patients (4.7%) had intolerance or severe side effects and 6 patients did not take imatinib for economic reasons. Among the 95 patients with tolerance of imatinib, 18 patients (18.9%) interrupted treatment for side effects, such as diarrhea, constipation, and low leucocyte amount. However, the 18 patients recovered soon after stopping imatinib for one or more months, while 14 patients continued to take imatinib until the time was sufficient. The mean duration of follow-up, calculated from the data collection closure (March 2017), was 39 months (interquartile range [IQR], 23–59 months). The 5-year RFS rates and 5-year overall survival (OS) rate of all of patients were 36% and 78%, respectively.

**Table 1:** Baseline characteristics of 185 patients with high-risk GISTs.

Characteristics	All patients	Group A	Group B	Group C	Group D	*P* value
Age (M ± SD, years)	54.8 ± 12.0	56.9 ± 12.8	53.7 ± 9.9	52.5 ± 10.5	52.1 ± 13.1	0.13
Gender (M/F, n)	77/108	39/51	15/25	15/15	8/17	0.53
Tumor size (cm)	8.6 ± 5.8	8.9 ± 6.3	7.9 ± 4.9	9.8 ± 7.1	7.2 ± 2.0	0.32
Mitotic (/50 HPF)						0.33
<5	36	21	6	4	5	
5–10	96	23	13	13	4	
>10	53	46	21	13	16	
Tumor sites (n)						0.64
Gastric	124	60	28	20	16	
Small bowel	49	21	11	9	8	
Colorectal	12	9	1	1	1	
GI bleeding (Y/N, n)	116/69	35/55	12/28	11/29	15/10	0.11

M ± SD: mean ± standard deviation.

GI bleeding: gastrointestinal bleeding.

### Analysis and statistics of RFS and OS

**Figure 1:**
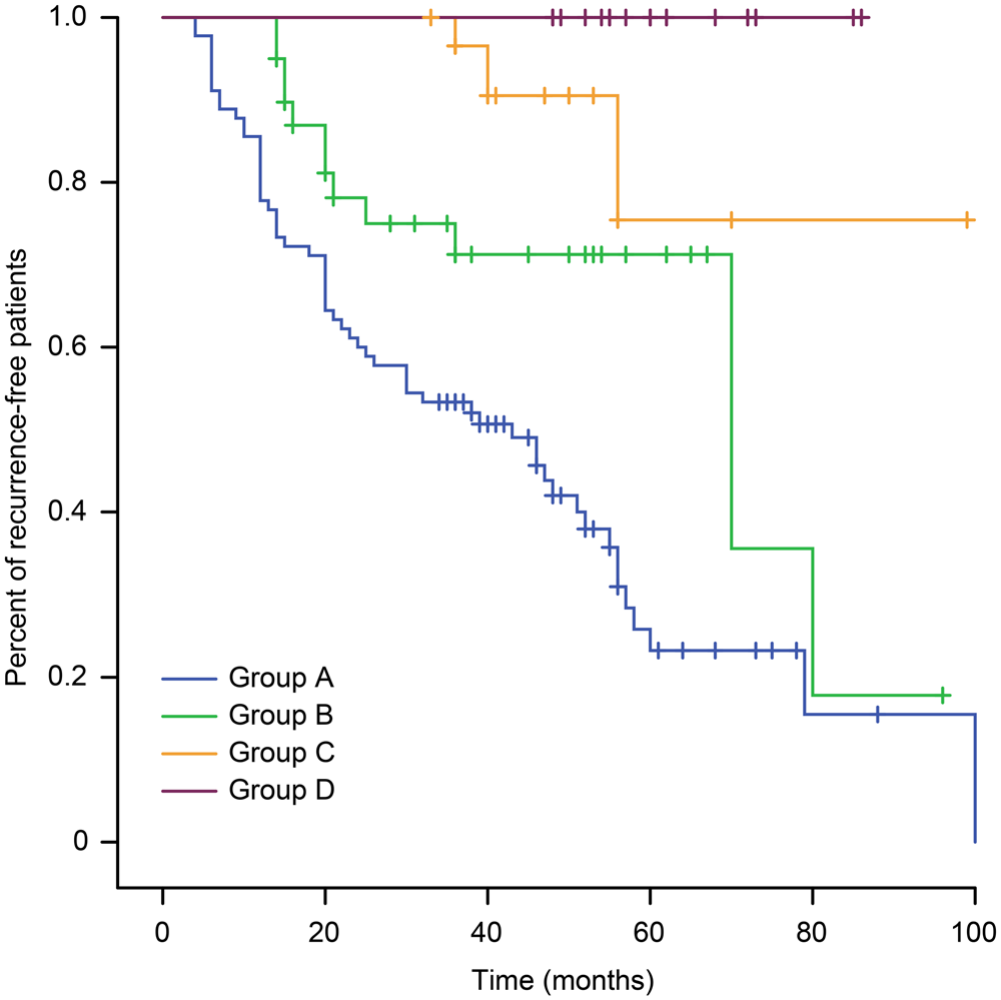
Kaplan–Meier estimates of the RFS of 185 patients in different groups.

**Figure 2:**
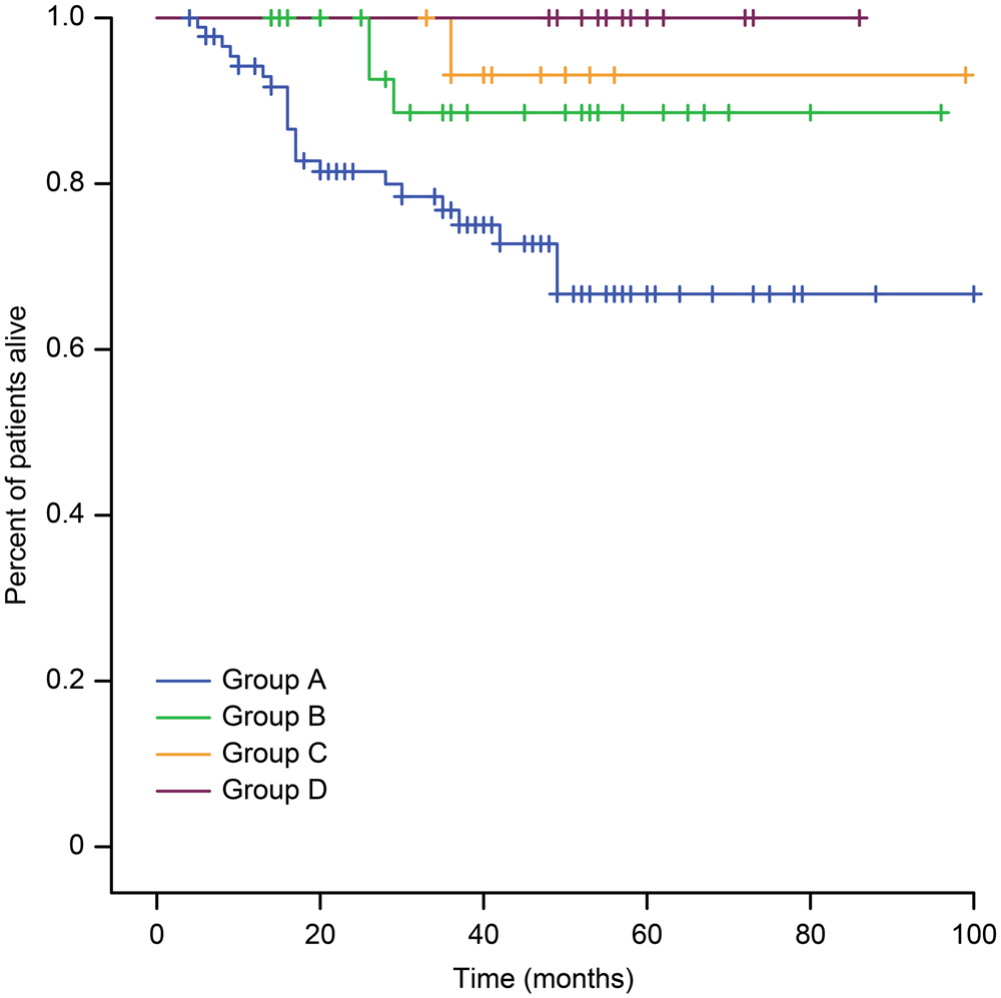
Kaplan–Meier estimates of the OS of 185 patients in different groups.

We used the Kaplan–Meier method and log-rank test model to examine the duration of the adjuvant imatinib effect on RFS and OS in different groups. The mean RFS among all patients was 62.8 (95% CI, 56.5–69.1) months and the OS was 86.3 (95% CI, 81.4–91.0) months. The means of RFS in Groups A, B and C were 44.3 (95% CI, 36.5–52.0), 62.1 (95% CI, 50.5–73.8), and 86.8 (95% CI, 73.3–100.3) months, respectively (*P* < 0.001) (Fig. 1). The means of OS in these groups were 75.2 (95% CI, 66.4–84.0), 88.1 (95% CI, 79.7–96.5), and 94.7 (95% CI, 88.8–100.5) months, respectively (*P* = 0.009) (Fig. 2). None of the patients in Group D had recurrence; thus, the mean of PFS or OS could not be calculated. However, the 5-year RFS rates in Groups A, B, C, and D were 15%, 26%, 83%, and 100%, respectively (*P* < 0.001) (Fig. 1). The 5-year RFS of Group A was lower than that of Group B (*χ* ^*2*^ = 7.66 *P* = 0.006), and it was significantly different between Group B and Group C (*χ* ^*2*^ = 5.32 *P* = 0.02). The 5-year RFS of Group D was higher than that of Group C (*χ* ^*2*^ = 4.73 *P* = 0.03) (Fig. 1). The 5-year OS rates the groups were 64%, 88%, 88%, and 100% (*P* < 0.001), respectively (Fig. 2). The 5-year OS rate of Group A was lower than that of Group B (*χ* ^*2*^ = 4.54 *P* = 0.033), but it was insignificantly different between Groups C and B (*χ* ^*2*^ = 0.53 *P* = 0.47) and between Groups C and D (*χ* ^*2*^ = 1.76 *P* = 0.19). Group B vs. Group D did not show any significant difference (*χ* ^*2*^ = 2.97 *P* = 0.09) (Fig. 2).

### Cox proportional hazards model of the prognostic factors

In our study, death as a result of GISTs during our follow-up was minimal and could interfere with the accuracy of the statistics of OS, since the Cox proportional hazards model analyzed only multiple prognostic factors associated with the recurrence of high-risk patients with GISTs. To reduce the bias, we divided the duration of adjuvant imatinib, tumor size, tumor mitotic, tumor site and GI bleeding into different levels, as shown in Table 2. Through the multivariate analyses of risk factors with GISTs recurrence, GI bleeding was not associated with outcomes of high-risk patients with GISTs (*P* = 0.43) (Table 3). Tumor site was a risk factor for GIST recurrence (HR, 1.87, 95% CI, 1.13–3.10; *P* = 0.01) (Table 3), and tumor size was also associated with unfavorable outcomes (HR, 1.06, 95% CI, 1.02–1.11; *P* = 0.002) (Table 3). The greatest impact on unfavorable outcomes of high-risk patients with GISTs was the tumor mitotic rate (HR, 2.01, 95% CI, 1.38–2.94; *P* < 0.001) (Table 3). Duration of adjuvant imatinib was the only favorable factor with RFS (HR, −1.95, 95% CI, 1.93–1.97; *P* < 0.001) (Table 3).

**Table 2:** Different levels of prognostic factors.

	Patient and Tumor		
Factor	No.	(%)	Levels
Duration of adjuvant imatinib (m)			
0–11	90	48	0
12–24	40	22	1
25–36	30	16	2
37–48	3	2	3
49–60	15	8	4
>60	7	4	5
Tumor size (cm)			
≤5.0	56	30	1
5.1–10.0	78	42	2
10.1–15.0	37	20	3
15.0–20.0	10	7	4
>20	4	2	5
Tumor mitotic rate (count per 50 HPFs)			
≤5	32	17	1
6–10	53	28	2
11–15	36	20	3
16–20	27	15	4
>20	37	20	5
Tumor site			
Stomach	124	67	0
Small intestine	49	27	1
Colon or rectum	12	6	1
GI bleeding			
Yes	116	63	1
No	69	37	0

**Table 3:** Multivariate analyses of risk factors for RFS.

Covariates	HR	95%CI		P
GI bleeding	1.24	0.73	2.11	0.43
Tumor mitotic rate	2.01	1.38	2.94	<0.001
Tumor site	1.87	1.13	3.10	0.01
Tumor size	1.06	1.02	1.11	0.002
Duration of adjuvant imatinib	−1.95	1.93	1.97	<0.001

HR: hazard ratio.

## Discussion

The incidences of gastrointestinal stromal tumors (GISTs) have been increasing^1,27,28^. Surgery and adjuvant imatinib are the most important treatment for high-risk patients with GISTs. Imatinib, a small-molecule inhibitor of these tyrosine kinases, is the recommended first-line option for metastatic and unresectable GISTs according to the Clinical Practice Guidelines in 2011^29,30^. However, the duration of adjuvant imatinib for high-risk patients who underwent complete resection has remained controversial.

Our retrospective study showed that the 5-year RFS rates in Groups A, B, C and D were 15%, 26%, 83%, and 100%, respectively (*P* < 0.001) (Fig. 1). The RFS of Group C was more favorable than that of Group B (*P* = 0.02), which was in line with previous studies^18^. Additionally, Group D was better than Group C (*P* = 0.03), which was the same as another retrospective study^31^. Based on previous studies and our retrospective data, adjuvant imatinib for three years was not sufficient for high-risk patients with GISTs, and a longer duration of adjuvant imatinib might decrease recurrence. In 2017, results from PERSIST-5 study reported by the ASCO conference indicated that 5-year adjuvant therapy may further prolong disease-free survival of intermediate and high risk patients. However, there were two main factors restricting long-term treatment. One was the side effects associated with imatinib, and another was the resistance of imatinib. Although clinical experience with imatinib demonstrated that many side effects were mild compared with other chemotherapy or targeted drugs, many studies reported that all patients treated with imatinib had at least one adverse event of any grade, and 21–43% experienced one or more Grade 3 or 4 adverse events at the 400 mg daily dose^10,11,13,32^. Thus, for a long duration of imatinib, a large proportion of patients had clinically significant toxicity, and treatment interruptions were not uncommon; these might ultimately alter the efficacy. The resistance of imatinib could be divided in two categories. Firstly, patients who progress within 6 months of an initial clinical response had primary resistance caused by an over-represented *KIT* exon 9 mutation or no detectable kinase mutation (wild-type tumor) in tumors^6,33–35^. The primary resistance to imatinib was minimal, but secondary resistance eventually developed in the majority of patients with imatinib^36,37^. The average time of secondary resistance was 20 months after treatment with imatinib^38^. Secondary resistance was found in 50–70% of the patients showing late progression. Secondary resistance occurred not only in late progression but also in the early progression, which had a great impact on the long duration of adjuvant imatinib for high-risk patients. Although knowledge about the mechanisms of resistance to imatinib has increased rapidly in recent years, it is unlikely that the whole spectrum of resistance mechanisms have now been elucidated; this has great importance, as it forms the foundation for treatment individualization and for the development of novel treatment approaches^39,40^. The optimal duration and scheduling of imatinib was unclear and 3 years was not sufficient for high-risk patients with GISTs. More research is still needed for the management of imatinib. Now, many researchers are exploring the optimal duration of adjuvant imatinib. For example, ‘Efficiency of Imatinib Treatment Maintenance or Interruption After 3 Years of Adjuvant Treatment in Patients with Gastrointestinal Stromal Tumors’ (NCT02260505) is sponsored by Centre Leon Berard, an open-label, randomized, multicenter phase III study aiming to determine the clinical impact of maintaining imatinib treatment beyond 3 years in the adjuvant setting for high-risk patients with GISTs.

In our study, most of the relapsed patients were discovered earlier for regular follow-up and were treated in a timely manner, e.g., reoperation or high-dose imatinib instead of sunitinib. This led to less patient deaths with GISTs recurrence (5-year OS, 78%), lower than that reported by other studies^14,17,18,41^. This suggests that regular follow-up is very important for high-risk patients with GISTs and can decrease the death rate for GISTs recurrence.

In the Cox proportional hazards model, duration of adjuvant imatinib was the only favorable factor with outcomes of high-risk patients with GISTs (*P* < 0.001) (Table 3). In 2012, contour maps were estimated by the risk of GIST recurrence after surgery by *Joensuu H* and are appropriate for estimation of individualized outcomes^42–44^. The high-risk GISTs classified by the modified NIH criteria were distributed into 30–100% of the 10-year risk of GIST recurrence in contour maps, indicating that the outcomes of high-risk GISTs had vast differences. Therefore, durations of adjuvant imatinib should be different for the same high-risk patients with GISTs. Adjuvant imatinib for three years might be enough for some patients, while it should be prolonged to decrease recurrence for others. Non-gastric GISTs (intestine and colon) were unfavorable compared with gastric GISTs^20,45,46^. Attending physicians should advise patients suffering non-gastric GISTs to prolong the duration of adjuvant imatinib.

In our data, the most important independent factor associated with an unfavorable outcome was the tumor mitotic rate (*P* < 0.001) (Table 3). When arranging the follow-up and planning duration of adjuvant imatinib, we should attach greater importance to high tumor mitotic rate than large tumor size. Even for the high tumor mitotic rate of median-risk GISTs, we should consider adjuvant imatinib for a short time. For patients with a high tumor mitotic rate, the follow-up after adjuvant imatinib might be arranged more frequently than for those with a low tumor mitotic rate.

Our retrospective study also found another interesting result about GI bleeding. GI bleeding was one of the most common presentations among patients with high-risk recurrence. However, whether GI bleeding indicates tumor rupture is unknown. From the Cox proportional hazards model of our data, we found that GI bleeding was not associated with the outcome of high-risk patients (*P* = 0.43) (Table 3). Some studies have reported that GI bleeding was an independent predictor of unfavorable prognosis^47,48^. However, some studies showed that GI bleeding did not seem to be associated with risk of recurrence^49^. A study reported that GI bleeding was a protective factor for GIST relapse and GI-bleeding patients who achieved a better prognosis^50^. It remains controversial whether GI bleeding was an independent predictor of unfavorable prognosis for patients with GISTs, and more comprehensive studies are needed.

There are certain limitations in our study. First, this is a retrospective study with a limited sample size and a large proportion of censored cases. Second, the study was conducted in one single central hospital. Third, mutational analysis of our data was incomplete before 2010, causing the lack of data on mutational status. Meanwhile, assessment of imatinib pharmacokinetics was used for all patients, and the lack of data was a limitation of our study. Finally, the short follow-up time might cause bias. Thus, a larger scale, multicenter, prospective study with a longer-term follow-up investigation is warranted. Despite these caveats, it appears that our findings can contribute to the management of imatinib in high-risk patients who underwent complete resection.

In summary, based on existing studies and our retrospective findings, we recommend that at least 3 years of adjuvant imatinib be standard care for high-risk patients with GISTs. For high-risk patients with a large tumor size, high tumor mitotic rate or non-gastric GISTs, we recommend that more than 3 years of adjuvant imatinib is also feasible but only if patients can tolerate the imatinib for a long duration. Individualized adjuvant imatinib for patients with GISTs and management of followup should be an important direction for future treatment of GISTs.

## Methods

### Patients

In this study, the data were retrospectively collected from 658 patients who underwent surgery for GISTs in West China Hospital, Sichuan University, from December 2009 to January 2015. The inclusion criteria were as follows: patients with *KIT* (CD117) positive in immunohistochemistry, patients without preoperative chemotherapy or imatinib, patients with high-risk GISTs confirmed by pathology, patients with complete resection and without distant metastasis, and patients beginning adjuvant imatinib between one and eight weeks after surgery. We used the modified NIH criteria as a risk-stratification scheme for GISTs; this has been generally accepted in clinical practice^51–53^. High-risk GISTs must meet at least one of the following features: maximum tumor diameter longer than 10.0 cm, mitotic count more than 10 mitoses per 50 high-power fields, tumor diameter above 5.0 cm and mitotic count over 5, and tumor rupture before surgery or during surgery. However, patients with tumor rupture were not included in our study because it might indicate incomplete resection^36^. The exclusion criteria were as follows: patients with other malignant diseases and patients who did not sign informed consent. By March 2017, we lost touch with 62 patients during the follow-up. Finally, after screening, a total of 185 patients were enrolled in the study from a total of 596 patients. The duration of adjuvant imatinib only included the time of patients receiving imatinib. The time, patients stopping imatinib for intolerance and side effects were not included. The dose of imatinib in patients who were tolerable (174 patients) was 400 mg qd, while 11 patients did not receive imatinib for 12 months, and 79 patients never took imatinib after surgery because of intolerance, side effects or other reasons. All patients were divided into 4 groups: <1 year (Group A, including 0–11 months, n = 90), 1–2 years (Group B, including 12–24 months, n = 40), 2–3 years (Group C, including 25–36 months, n = 30) and >3 years (Group D, including more than 36 months, n = 25) according to the duration of adjuvant imatinib. The primary endpoint was RFS and OS of the four groups. The risk recurrent factors of GISTs in our study were duration of adjuvant imatinib, tumor mitosis, tumor size, tumor site and gastrointestinal bleeding (GI bleeding), which was recently described in a study^48,54–56^. Through the results, we aimed to discover the appropriate duration of adjuvant imatinib for high-risk patients with GISTs.

### Follow-up

Professional researchers followed up with the postoperative patients once every 1–3 months within 2 years and once every 3–6 months in the 3–5 year period after surgery via telephone calls and outpatient service. Contrast enhanced computed tomography (CT) or magnetic resonance imaging (MRI) of the abdomen and pelvis and CT of the chest or chest X-ray were required with the first dose of imatinib. The patients had CT or MRI of the abdomen and pelvis at 3- to 6-month intervals during the follow-up. Blood cell counts and chemistries were performed at 1- to 3-month intervals during the treatment period and subsequently at 6-month intervals after stopping imatinib.

### Ethics statement

The study protocol was approved by the ethics committee of West China Hospital, Sichuan University. Written informed consent was obtained from the patients before beginning the study, even though the study is retrospective nature. The analysis did not involve interaction with human subjects or the use of personal identifying information. Patient records/information was anonymized and de-identified prior to analysis, and the methods were performed in accordance with the approved guidelines.

### Statistical analyses

All statistical analyses were tested using SPSS version 20.0 (for MAC, IBM). We used the *χ* ^*2*^ test to compare categorical data and the *t* test or ANOVA to compare continuous data. The survival rate was compared using the Kaplan–Meier method, and the log-rank test model was used to detect differences in the survival curves of the various subgroups. The Cox proportional hazards model was used to analyze the prognostic factors of multiple factors associated with RFS. All *P* values were 2-sided, and *P* values < 0.05 were considered significant.
